# Phenotypic overlap between atopic dermatitis and autism

**DOI:** 10.1186/s12868-021-00645-0

**Published:** 2021-06-22

**Authors:** Kyong-Oh Shin, Debra A. Crumrine, Sungeun Kim, Yerin Lee, Bogyeong Kim, Katrina Abuabara, Chaehyeong Park, Yoshikazu Uchida, Joan S. Wakefield, Jason M. Meyer, Sekyoo Jeong, Byeong Deog Park, Kyungho Park, Peter M. Elias

**Affiliations:** 1 Department of Food Science/Nutrition, & Convergence Program of Material Science for Medicine/Pharmaceutics, and the Korean Institute of Nutrition, Hallym University, Chuncheon, South Korea; 2 Dept. of Dermatology, University of California, NCIRE, and Veterans Affairs Medical Center, 4150 Clement Street, MS 190, San Francisco, CA 94121 USA; 3 Department of Dermatology, University of San Francisco, San Francisco, CA USA; 4 Dept of Cosmetic Science, Seowon University, Cheongju, South Korea; 5 Sphingobrain Inc., San Francisco, CA USA; 6 Dr. Raymond Laboratories, Inc, Englewood Cliffs, NJ USA

**Keywords:** Atopic dermatitis, Autism, Autism spectrum disorders, Blood–brain barrier, IFNγ, IL-4, 5, 13 and 17A, TNFα, Valproic acid mouse model

## Abstract

**Background:**

Autism, a childhood behavioral disorder, belongs to a large suite of diseases, collectively referred to as autism spectrum disorders (ASD). Though multifactorial in etiology, approximately 10% of ASD are associated with atopic dermatitis (AD). Moreover, ASD prevalence increases further as AD severity worsens, though these disorders share no common causative mutations. We assessed here the link between these two disorders in the standard, valproic acid mouse model of ASD. In prior studies, there was no evidence of skin involvement, but we hypothesized that cutaneous involvement could be detected in experiments conducted in BALB/c mice. BALB/c is an albino, laboratory-bred strain of the house mouse and is among the most widely used inbred strains used in animal experimentation.

**Methods:**

We performed our studies in valproic acid (VPA)-treated BALB/c hairless mice, a standard mouse model of ASD. Mid-trimester pregnant mice received a single intraperitoneal injection of either valproic acid sodium salt dissolved in saline or saline alone on embryonic day 12.5 and were housed individually until postnatal day 21. Only the brain and epidermis appeared to be affected, while other tissues remain unchanged. At various postnatal time points, brain, skin and blood samples were obtained for histology and for quantitation of tissue sphingolipid content and cytokine levels.

**Results:**

AD-like changes in ceramide content occurred by day one postpartum in both VPA-treated mouse skin and brain. The temporal co-emergence of AD and ASD, and the AD phenotype-dependent increase in ASD prevalence correlated with early appearance of cytokine markers (i.e., interleukin [IL]-4, 5, and 13), as well as mast cells in skin and brain. The high levels of interferon (IFN)γ not only in skin, but also in brain likely account for a significant decline in esterified very-long-chain *N*-acyl fatty acids in brain ceramides, again mimicking known IFNγ-induced changes in AD.

**Conclusion:**

Baseline involvement of both AD and ASD could reflect concurrent neuro- and epidermal toxicity, possibly because both epidermis and neural tissues originate from the embryonic neuroectoderm. These studies illuminate the shared susceptibility of the brain and epidermis to a known neurotoxin, suggesting that the atopic diathesis could be extended to include ASD.

**Supplementary Information:**

The online version contains supplementary material available at 10.1186/s12868-021-00645-0.

## Background

The prevalence of autism spectrum disorders (ASD) is approaching 2% in populations of all racial, ethnic and socioeconomic groups, with a fourfold male predominance (https://www.tacanow.org/family-resources/latest-autism-statistics-2/ and https://www.cdc.gov/ncbddd/autism/data.html). Autism is diagnosed when a patient demonstrates deficits in social interactions; a lack of social or emotional reciprocity; and learning disabilities [1]. While the diagnosis of ASD is not firmly established until 24 months of age, abnormalities in fMRI scans; preferences for geometric patterns; aberrant social communications; changes in fine motor skills; and/or characteristic eye movements can often be detected as early as 6 months of age [2, 3].

Although over 100 genes are now associated with ASD [4], neonatal exposure to environmental risk factors such as microbial pathogens or certain drugs (e.g., the neurotoxin, valproic acid [VPA] [5]), can also provoke ASD [6, 7]. VPA induces broad abnormalities in genes that regulate cell cycle, cell wall biogenesis, DNA repair and ion homeostasis [8, 9]. In fact, VPA has traditionally been prescribed to control epilepsy [10], or for treatment of psychiatric conditions, such as bipolar disorders, through its modulation of GABA neurotransmission [11]. Since cumulative epidemiological and clinical studies have shown that prenatal exposure to VPA is tightly linked to a significant increase in the risk of ASD; i.e., the rate of ASD in the children of VPA-exposed mothers is approximately eight times higher than that of the general population [12, 13], in utero exposure of rodents to VPA has also been proposed as a robust animal model of ASD. This murine model shows great similarities to human features of ASD, including three core deficits: (i) impaired reciprocal social interaction; (ii) restricted, repetitive and stereotyped patterns of behaviors or interests, and (iii) communication deficits, likely reflecting common neuronal alterations in ASD. VPA also inhibits histone deacetylase (HDAC) [14], epigenetically modifying histone H3 and H4, in turn activating the histone acetyltransferase transformation/transcription domain-associated protein (TRRAP) [15]. Yet, several other genes (e.g., Sox10, Pdgfra, Plp, and Cnp) that regulate axon development, which are first expressed at day 12.5 [16], could also represent targets of VPA. The net results in exposed animals and humans include early axonal overgrowth and increased network excitability [6] (Additional file 1: Fig. S1).

These changes in neuronal structure and function correlate with neuropathologic alterations that largely localize to the somatosensory cortex [17]. Patients typically display a marked increase in synaptic density [18]; decreased thickness of myelin sheaths; increased axon branching; and reductions in white matter water content [19–22]. While in normal infants, the number of synapses increases up to 2 years of age, and subsequently declines, ASD is characterized by a failure of this downstream ‘pruning’ process [18].

Though it has been proposed that these structural alterations reflect maternal immune activation (MIA) [23–25], this pathogenic link is still unclear, because evidence of neuroinflammation is often lacking in ASD. The MIA theory further proposes that neuroreactive Th-1 and antigen-specific Th-17 cells [25, 26] traverse the infant’s blood–brain barrier (BBB), which is characteristically immature during infancy [27]. These cytokines further stimulate the release of mast cell mediators, purportedly augmenting neuroinflammation [28], perhaps further compromising the BBB [29]. Among the invading cytokines, IL-17A could be particularly important in ASD pathogenesis, because it attacks both neurons and oligodendrocytes [27].

Atopic dermatitis (AD) and other atopic disorders exhibit a strong association (≈ 10%) with ASD [30–37], and a higher prevalence as AD phenotypes worsen [38, 39]. Yet, despite the long-appreciated association of ASD with AD, the basis for the link between these two diseases has not been explored. Though ASD is associated with numerous inherited mutations [4, 30], none are shared with the common inherited abnormalities that underlie AD, which instead compromise proteins that normally sustain epidermal structure and function [rev. in 40. In searching elsewhere for clues about the possible link between AD and ASD, we noted two underappreciated facts—first, that the epidermis and central nervous system (CNS) share a common embryologic origin in the primitive neuroectoderm [rev. in 41]; and second, not only the BBB [27], but also the permeability barrier displays suboptimal competence during the perinatal period [42].

Hence, we hypothesized first, that the shared embryologic origin of the brain and epidermis could render both tissues susceptible to common insults, which could explain, in turn, the shared baseline association of AD and ASD. If true, neurotoxins that provoke ASD should also preferentially attack the epidermis. Moreover, because insults that further compromise the already suboptimal cutaneous permeability barrier of neonates should further stimulate production of multiple, epidermal-derived cytokines [43, 44], it then seems plausible that epidermal-derived, pro-inflammatory cytokines, released in response to toxin-induced insults to the epidermis, could enter the circulation, traverse the infant’s immature BBB, initiating or amplifying neuroinflammation. Pertinently, neonatal tissues, including the skin, normally generate innate immune markers, as well as abundant Th1- and Th2-type cytokines [45, 46], likely generated to protect against colonization from perinatal exposure to pathogens [47]. Moreover, cutaneous cytokine production increases further as newborn skin becomes exposed to a xeric external environment [48]. Thus, increased cutaneous cytokine production due to the co-vulnerability of brain and epidermis to in utero exposure to toxins [49] could first reach the circulation and breach the BBB, initiating/amplifying neuroinflammation in ASD. Pertinently, the sustained permeability barrier abnormality in aged skin [50, 51] stimulates the generation of three key, age-related cytokines (IL-6, IL-1β, tumor necrosis factor [TNF]α) [52, 53] that reach the circulation [54], accounting for the aging ‘inflammasome’ [55].

To explore the possible basis for the AD-ASD phenotypic overlap, and the contribution of the skin to the provocation or exacerbation of AD-associated ASD, we assessed the chronology of structural, functional, lipid biochemical, and inflammatory changes in the skin and brain as they emerged in neonatal offspring of valproic acid (VPA)-exposed, mid-trimester pregnant mice [56, 57], a model that already has provided important insights into ASD pathogenesis [14, 58, 59]. VPA is an anti-epileptic drug that is still widely prescribed for women of child-bearing age who have epilepsy, though its use is associated with an increased risk of congenital malformations and impaired cognition. In hairless mice, it was readily apparent that not only neurotoxicity, but also previously-unrecognized epidermal cytotoxicity is present at birth, accompanied by high levels of cytokine indicators of toxicity/chronic inflammation (i.e., TNFα, IFNγ, and IL-17A), as well as markers of allergic (th2)-type inflammation in both the skin and brain (Table 1). We show here further that characteristic AD-like, lipid biochemical features [60], likely induced by elevated IFNγ levels [61–63], appear not only in the epidermis [64, 65], but also in the brain. Together, these results explain the phenotypic overlap of these two disorders, while also supporting a new paradigm for disease pathogenesis in the subset of ASD patients associated with AD. Herein, we explore the mechanistic basis for the AD-ASD association in the VPA mouse model.

**Table 1:**
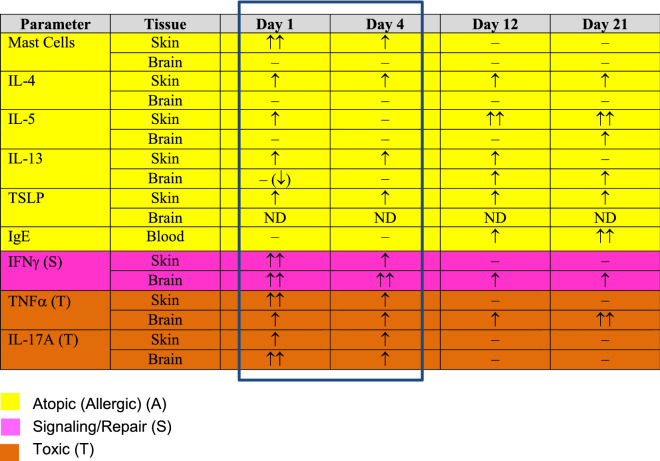
Sequential appearance of inflammatory markers in skin and brain of VPA-exposed neonatal mice

All data shown in Table indicate values in ASD mice compared to vehicle-treated mice.

↑: increased; ↓: decreased; ↑↑: greatly increased; –: not altered; ND: not detected

## Methods

### Animals and housing conditions

All animal procedures were approved by the Institutional Animal Care and Use Committee (IACUC) of Hallym University (Permit number: Hallym-2018-84) and performed in accordance with their guidelines as well as ARRIVE guidelines (Animal Research: Reporting of In Vivo Experiments) (https://www.nc3rs.org.uk/arrive-guidelines). Nine-week-old pregnant BALB/cJ female mice, weighting 25.4 (mean) ± 2.4 g, were purchased from DBL Ltd. (Eumseong, Korea). All experimental mice were housed in individual cages at the Hallym University Laboratory Animal Resources Center under specific pathogen-free (SPF) conditions with a controlled consistent temperature (23 ± 2 °C) and lighting environment (12 h/12 h light/dark cycle). Mice were fed with the standard irradiated chow diet (Purina, Seongnam, Korea) for rodent ad libitum and drinking water. At the end of the study, the experimental mice were sacrificed by CO_2_ inhalation. A gradual fill rate of 20% chamber volume per minute displacement was used for CO_2_ euthanasia. All efforts were made to minimize the number and suffering of any animals used in these experiments.

### Experimental protocols

Twenty pregnant BALB/c mice were randomly divided into two experimental groups, and were treated intraperitoneally with either 600 mg/kg valproic acid sodium salt (VPA, Sigma, MO, USA) dissolved in saline (n = 12) or saline alone (n = 8) on embryonic day 12.5 (E12.5). VPA- or saline (vehicle)-injected mice were housed in individual cages, and pups from VPA- or vehicle-treated dams were maintained until up to postnatal days 21 (P21). While 41 pups from VPA-treated dams and 59 pups from vehicle-treated dams were employed in this study, both brain and skin tissues for downstream assays were obtained from pups of VPA- or vehicle-treated dams on P1 (n = 9 or n = 13, respectively), P4 (n = 11 or n = 12, respectively), P12 (n = 9 or n = 15, respectively) and P21 (n = 12 or n = 19, respectively). The same experiment was repeated using 22 pregnant mice. 39 pups from VPA-treated dams and 47 pups from vehicle-treated dams were used in the 2nd batch of experiments, and brain/skin tissues were obtained from VPA- or vehicle-exposed pups on P1 (n = 9 or n = 11, respectively), P4 (n = 8 or n = 9, respectively), P12 (n = 9 or n = 10, respectively) and P21 (n = 13 or n = 17, respectively).

### Epidermal functional studies

Prior to performing epidermal functional studies, mice (pups) were anesthetized with 2% isoflurane in a combination of nitrous oxide and oxygen (7:3, v/v) via an isoflurane vaporizer (VetEquip, Livermore, CA). Basal epidermal permeability barrier function was assessed by measuring transepidermal water loss (TEWL) using TM300 connected to MPA5 (C&K, Cologne, Germany) between 10:00 a.m. and 12:00 p.m. during the light phase of the circadian cycle, as described previously [55]. In addition, epidermal permeability barrier function was assessed qualitatively by toluidine blue staining, as reported previously [66]. Briefly, 4-day old pups from both VPA- and vehicle-treated dams were euthanized and fixed in methanol at room temperature. After washing five times with PBS, mice were incubated with 0.1% toluidine blue solution dissolved in saline, followed by washing with PBS, then mice were examined and photographed for the extent of penetration of the blue dye into the skin.

### Behavioral study

Spatial learning and memory performance were assessed using the Morris water maze task, as described previously [67, 68] (Additional file 1: Fig. S1). Briefly, a 9 cm diameter platform was placed in the southeast quadrant of the 1.2 m diameter circular pool filled with a room-temperature water. After the completion of a training session which consisted of three trials, a visible trail, hidden-platform trial, and probe trial, for 4 days, the mice were given three trials for another 3 days to test their ability to locate a visual or hidden platform, or to evaluate the number of times/duration that treated mice crossed the hidden platform. Each trial was recorded with a ceiling-mounted video camera (Ganz YCH-02, Cary, NC, USA), and analyzed using automated tracking software (Ethovision XT 6, Noldus, Wageningen, Netherlands). The Morris water maze task was conducted between 09:00 a.m. and 16:00 p.m. during the light phase of the circadian cycle. An hour before the behavioral test, all experimental mice were transported from the housing room to behavioral testing rooms, and they were left to acclimatize to their new surroundings, as well as recover from any stress caused by the transportation. After completing the behavioral study, mice (pups) were anesthetized with 2% isoflurane in a combination of nitrous oxide and oxygen (7:3, v/v) via an isoflurane vaporizer (VetEquip, Livermore, CA) for the downstream experiments.

All procedures were subjected to approval by the Ethical Committee on Animal Experiments, Hallym University, Korea (permit number: Hallym-2018-84) and performed accordingly.

### Histological analyses

Prior to tissue preparation for histological analyses, mice (pups) were anesthetized with 2% isoflurane in a combination of nitrous oxide and oxygen (7:3, v/v) via an isoflurane vaporizer (VetEquip, Livermore, CA). The change in the overall morphology in skin and brain was assessed by hematoxylin and eosin staining, as described previously [69]. Distribution of tumor necrosis factor (TNF)α, interferon (IFN)γ, and interleukin (IL)-13 was determined using anti-TNFα, anti-IFNγ, or anti-IL-13 (Invitrogen, Carlsbad, CA), respectively, as described earlier [70]. The secondary antibody was Alexa Fluor® 488 goat anti-rabbit IgG (Invitrogen, Carlsbad, CA). Tissues were counterstained with the nuclear marker 4′,6-diamidino-2-phenylindole (DAPI) (Vector Laboratories) for nuclear visualization. Slides were examined with a Carl Zeiss Axio fluorescence microscope.

### Electron microscopy

Skin or brain biopsies from both VPA- and vehicle-treated mice were taken for electron microscopy after anesthetization with 2% isoflurane in a combination of nitrous oxide and oxygen (7:3, v/v) via an isoflurane vaporizer (VetEquip, Livermore, CA). Briefly, tissues were fixed in modified Karnovsky’s fixative overnight, and post-fixed in either 0.2% ruthenium tetroxide or 1% aqueous osmium tetroxide, containing 1.5% potassium ferrocyanide. After fixation, all tissues were dehydrated in a graded ethanol series, and embedded in an Epon-epoxy mixture. Ultrathin sections were examined, with or without further contrasting with lead citrate, in a Zeiss 10A electron microscope (Carl Zeiss, Thornwood, NJ), operated at 60 kV.

### ELISA for cytokine quantification

Blood, skin or brain biopsies from both VPA- and vehicle-treated mice were collected for cytokine quantifications after anesthetization with 2% isoflurane in a combination of nitrous oxide and oxygen (7:3, v/v) via an isoflurane vaporizer (VetEquip, Livermore, CA, USA). Levels of proinflammatory cytokines, e.g., IL-4, IL-5, IL-13, IL-17A, thymic stromal lymphopoietin (TSLP), IFNγ, TNFα, and IgE were quantitated using appropriated ELISA kits obtained from Thermo Fisher Scientific (Walthan, MA, USA) or Komabiotech (Seoul, South Korea) in accordance with the manufacturer’s instructions.

### Quantification of sphingolipids by liquid chromatography and tandem-mass spectrometry (LC–MS/MS)

Skin or brain biopsies from both VPA- and vehicle-treated mice were taken for sphingolipid quantifications after anesthetization with 2% isoflurane in a combination of nitrous oxide and oxygen (7:3, v/v) via an isoflurane vaporizer (VetEquip, Livermore, CA, USA). The levels of ceramide (Cer) and sphingomyelin (SM) were quantified using the LC–ESI–MS/MS (API 3200 QTRAP mass, AB/SCIEX) by selective ion monitoring mode, as described previously [70–72]. The MS/MS transitions of ceramides depending on their acyl chain length were 510 → 264 for C14-ceramide, 538 → 264 for C16-ceramide, 552 → 264 for C17-ceramide, 566 → 264 for C18-ceramide, 594 → 264 for C20-ceramide, 648 → 264 for C24:1-ceramide, and 650 → 264 for C24-ceramide, respectively. In addition, the sphingomyelin MS/MS transitions were 718 → 184 for C17 SM (d18:1/17:0) as an internal standard, 704 → 184 for C16 SM, 732 → 184 for C18 SM, 760 → 184 for C20 SM, 788 → 184 for C22 SM, 814 → 184 for C24:1 SM and 816 → 184 for C24 SM, respectively. Data were acquired using Analyst 1.5.1 software (Applied Biosystems, Foster City, CA). The results are expressed as pmol/mg protein.

### Statistical analyses

Data were expressed as the mean ± standard deviation (SD). Significance between groups was determined with unpaired Student t test. The P values were set at *P < 0.05.

## Results

### Provocation of a permeability barrier abnormality due to epidermal cytotoxicity in VPA-exposed neonatal mice

**Fig. 1:**
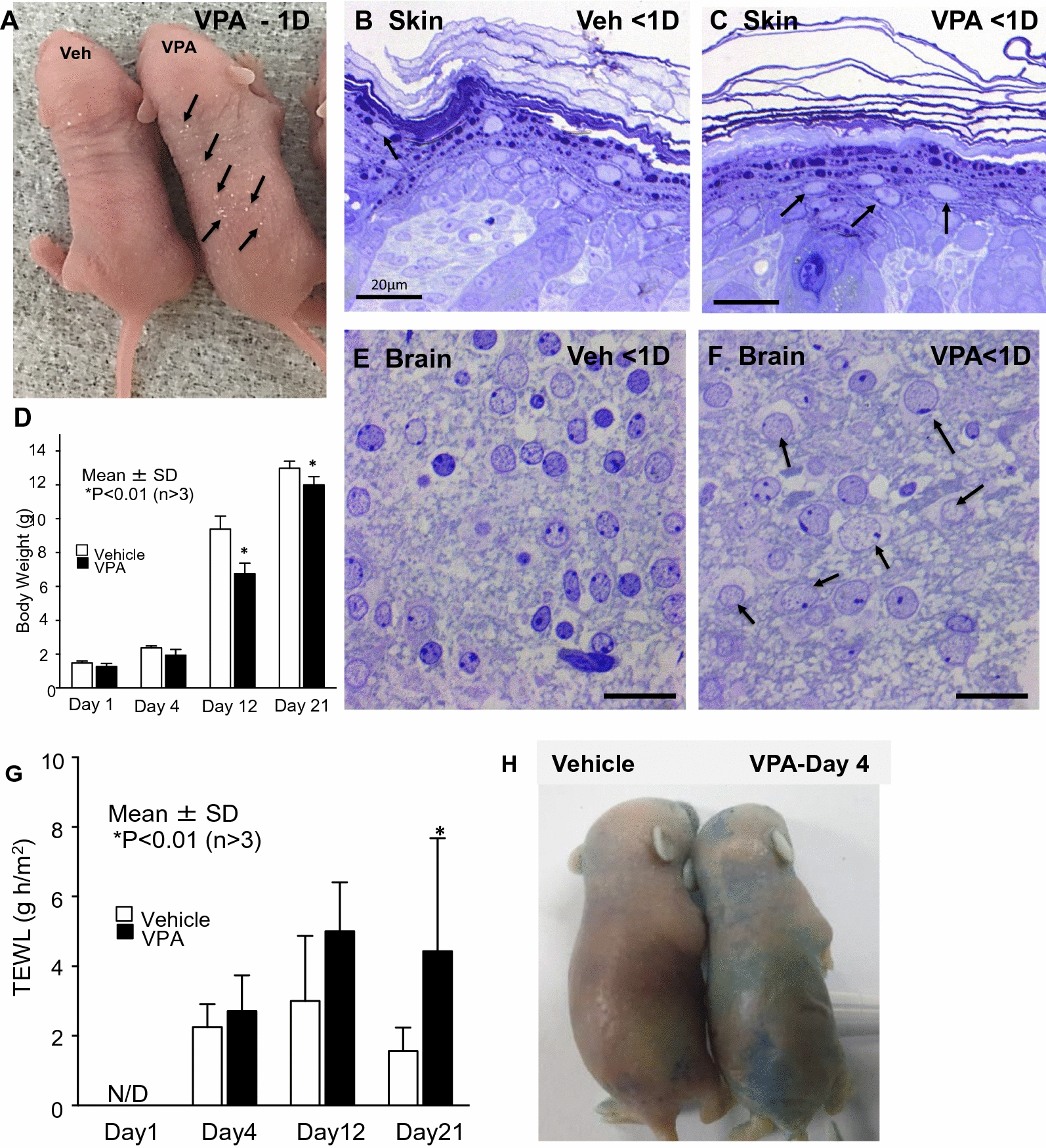
Valproic acid (VPA) exposure produces cytotoxicity in both skin and brain. Mid-trimester (12.5 days) pregnant BALB/c mice were injected with either VPA or saline vehicle (Veh). Tissue samples were assessed immediately after birth (< 1 day). **A** Generalized cutaneous scaling, with minimal inflammation was apparent at birth in VPA-exposed mice. **B**, **C** Cytotoxicity with minimal inflammation, evidenced as nuclear vacuolization in the outer epidermis of neonatal VPA-exposed mice (**C**, arrows). **D** Delayed weight gain in VPA-exposed mice. **E**, **F** Extensive toxicity, with ballooning degeneration of nuclei (arrows) in brains of VPA-exposed neonatal mice. **G** Quantitative assessment of barrier function as rates of transepidermal water loss (TEWL). **H** Leakage of toluidine blue into skin of 4-day-old VPA-exposed mice. **B**, **C**, **E**, **F** Mag bar = 20 μm

While the expected impact of VPA on the developing mouse brain was apparent at birth [56], prominent, previously unreported, cutaneous scaling also was immediately apparent in VPA-exposed, neonatal hairless mice (Fig. 1A). Shortly thereafter, characteristic behavioral abnormalities became evident, peaking at 7 days postpartum (Additional file 1: Fig. S1). Notably, a search for pathology in other organs revealed that only the epidermis and brain of these neonates demonstrated microscopic evidence of cytotoxicity (i.e., wide-spread vacuolization of cells in both the brain and outer nucleated layers of the epidermis (Fig. 1B, C, E, F). The finding of a selective onslaught by this neurotoxin on both the epidermis and the brain is supported by the common embryologic origin of these two tissues.

Evidence of cutaneous structural abnormalities was accompanied by defective cutaneous permeability barrier structure and function. Though neonatal VPA-exposed mice were too small for instrumental assessments of permeability barrier status, a subtle functional defect could be detected with an electron dense tracer, lanthanum nitrate (Additional file 1: Fig. S2), which serves as a surrogate measure of barrier status [e.g. 73]. This functional deficit became both more prominent and quantifiable at later time points (Fig. 1D), likely accounting for the animals’ reduced body weights from the additional caloric loss that accompanies excessive evaporative water loss. By 4 days postpartum, TEWL levels were significantly elevated in VPA-exposed mice vs. controls (Fig. 1G), while epicutaneous toluidine blue applications also demonstrated a diffuse permeability barrier abnormality (Fig. 1H).

These functional abnormalities correlated with structural defects throughout the epidermal lamellar body (LB) secretory system. Likely due to the above-noted, VPA-induced cytotoxicity, LBs did not form at the densities found in controls, and the quantities of lamellar material deposited at the stratum granulosum (SG)-stratum corneum (SC) interface were markedly reduced, paralleled by entombment of unsecreted lamellar body contents within the corneocyte cytosol (Additional file 1: Fig. S3A–C, open arrows). Post-secretory abnormalities, including a paucity of extracellular lamellar membranes, as well as prominent lamellar/non-lamellar phase separation, also were apparent (Additional file 1: Fig. S3B, asterisks). Notably, these structural and functional abnormalities mirror defects that have been described in both AD humans and murine models of AD [63–65].

### VPA-exposed skin and brain reveal lipid biochemical changes that mimic AD

**Fig. 2:**
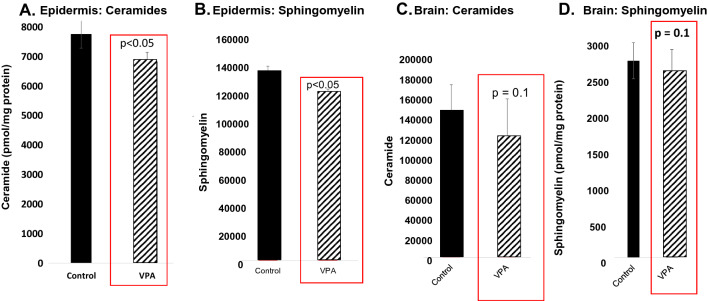

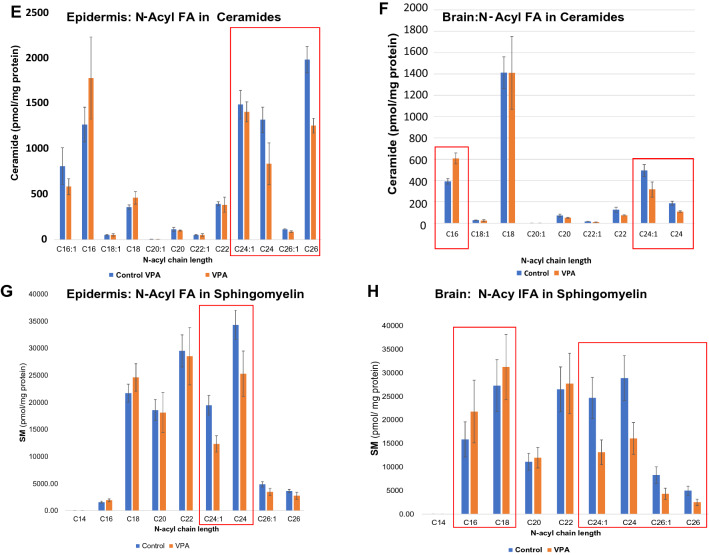
Decreased bulk sphingolipids and VLC *N*-acyl chain lengths in skin and brains of VPA-exposed mice. **A**, **B** Significant decline in content of both ceramides (Cer) and sphingomyelin (SM) in the skin of 1-day postnatal, VPA-exposed mice. **C**, **D** Similar decline in Cer and SM content in brains of similarly exposed mice. **E**–**H** Lipidomic analyses of *N*-acyl FA chain lengths in skin and brain sphingolipids in 1-day old, VPA- vs. vehicle-exposed mice

Epidermal lipids in both AD patients and in AD animal models consistently display marked declines in total ceramide (Cer) content [60]; as well as a concurrent shift from esterified, very-long chain *N*-acyl fatty acids (VLC-FA) towards shorter chain length species [63]. These changes have been attributed to both Th-2 cytokine-mediated downregulation of Cer production [74] and IFNγ-mediated downregulation of two fatty acid elongases; i.e., ELOVL1 and 4, respectively [62, 75]. Accordingly, we identified modest, but statistically significant parallel declines in both bulk Cer and sphingomyelin (SM) content in both the skin (Fig. 2A, B) and brain (Fig. 2C) of 1-day-old VPA-exposed mice. In parallel, the chain lengths of *N*-acyl fatty acids in both epidermal and brain Cer and SM shifted from VLC-FA towards shorter chain length species (Fig. 2E, F). Thus, by 1-day postpartum, both the skin and brains of VPA-exposed animals displayed changes in lipid composition that mirror AD (Ibid.).

### Cutaneous inflammation and neuroinflammation in VPA-exposed mice

**Fig. 3:**
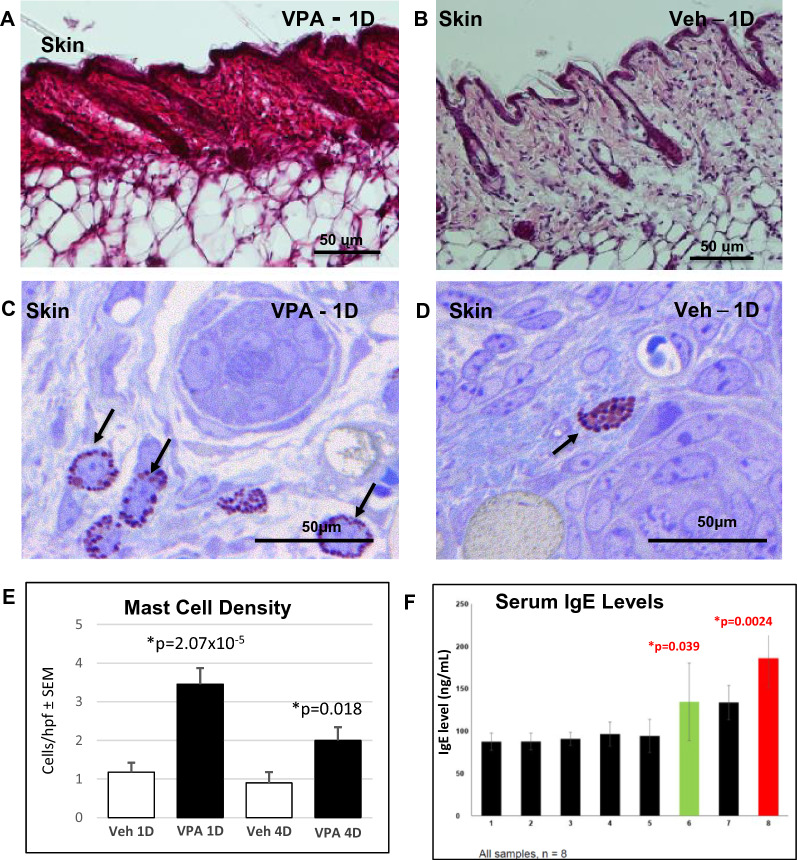
Cutaneous inflammation and mast cell hypertrophy precedes brain inflammation in VPA-exposed mice. **A**, **B** Dense cutaneous inflammatory infiltrate by day 1 in VPA-exposed mice (H + E staining). **C**, **D** Mast cell hypertrophy and degranulation at day 1 in toluidine blue-stained, one µm sections **E** Quantitation of mast cell density in VPA-exposed skin at days 1 and 4. **F** Changes in circulating IgE levels over time

AD and ASD characteristically appear in infancy. Hence, we next assessed inflammatory markers in VPA-exposed neonates. While both the skin and the brains of neonatal VPA-exposed mice displayed little evidence of toxicity and inflammation at birth, cutaneous inflammation became apparent by 1 day (Fig. 3A, B), but evidence of neuroinflammation was less evident at these early time points (Additional file 1: Fig. S4). By 1 day postpartum, allergic-type features began to emerge in the skin of VPA-exposed mice, reflected histologically by prominent mast cell hyperplasia and degranulation (Fig. 3C–E), features that were less apparent in VPA-exposed neonatal brains (not shown). Even at 4 days, destruction of microglia and oligodendrocytes was evident, with only minimal neuroinflammation (Additional file 1: Fig. S5). Together, these results illuminate the sequential emergence of cutaneous inflammation followed by neuroinflammation in VPA-exposed neonatal mice.

### Inflammatory markers in the skin and brain of VPA-exposed mice

**Fig. 4:**
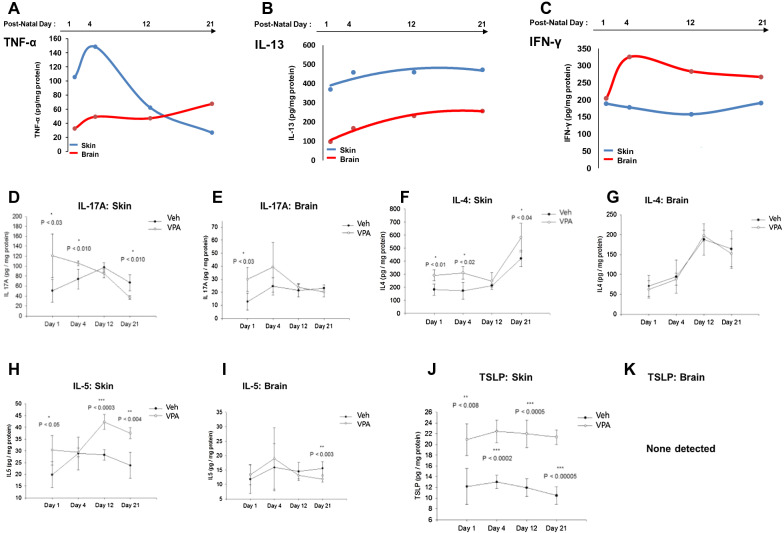
Cytokine profiles reflect concurrent skin/brain inflammation, with prior emergence of Th2 inflammation in skin (cf. Table 1). **A**–**D** Emergence of elevated TNFα, IFNγ, IL-13 and IL-17A in 1-day-old VPA- vs. vehicle (Veh)-exposed murine skin and brain; **B**, **E**–**F** higher levels of th-2 cytokines (IL-4, IL-5, IL-13) in 1-day old skin vs brain of VPA-exposed mice; **G** increased TSLP in skin, with absence of this pro-th2 cytokine in the brains of VPA-exposed mice

We next assessed evidence of *cutaneous* Th1 and Th2 inflammation [76, 77], as well as neuroinflammation, by evaluating the temporal emergence of pro-inflammatory cytokines in the skin and brain of neonatal VPA-exposed mice (Table 1). Although markers of toxicity and/or inflammation (i.e., elevated protein levels of TNFα, IFNγ, and IL-17A) were apparent at birth in both the skin and brain, the absolute levels of TNFα and IL-17A were much higher in neonatal skin than in the brain (Fig. 4A, D, and Additional file 1: Fig S4C–G). In contrast, IFNγ protein levels were comparably high in both tissues at birth, with brain levels exceeding skin levels soon thereafter (Fig. 4C; Additional file 1: Figs. S5A, B). Immunofluorescence images showed that these changes (including Th-2 cytokines—not shown) largely localize to the epidermis (Additional file 1: Fig. S6). For example, an increased signal for TNFα was observed at and just beneath the stratum corneum (SC)—stratum granulosum (SG) interface, while IFNγ immunolabeling was observed throughout the cytosol of suprabasal keratinocytes (Additional file 1: Fig. S6A–C), with peak staining at day 4 (Additional file 1: Fig. S6D).

**Fig. 5:**
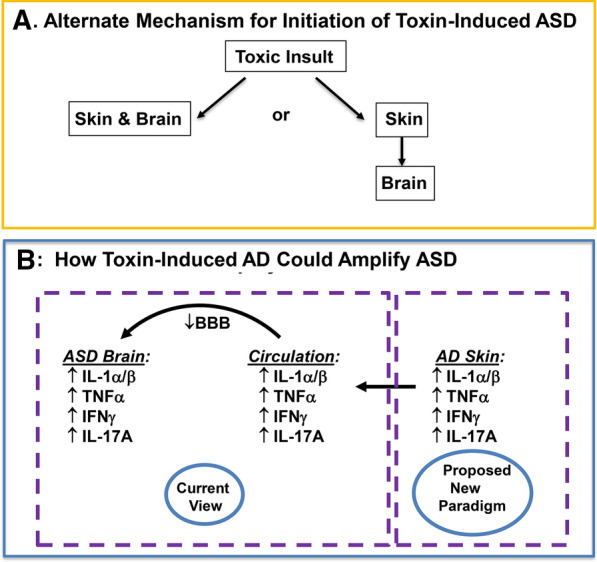
Diagrammatic summaries of potential skin-brain link in AD-associated ASD. **A**, **B** Proposed new paradigms for epidermal cytokine-driven neuroinflammation in AD-associated ASD

While markers of Th2-type inflammation (i.e., IL-4, IL-5, IL-13) were present at day one in both skin and brain, their magnitude was higher in the skin than in the brain (Table 1; Fig. 3F, M; Additional file 1: Fig. S5A–F). Protein levels for the same cytokines peaked at day 4 in the skin (Table 1; Figs. 4B, E, F; Additional file 1: Fig. S6C, D), and the increase in Th-2 cytokines again localized largely to the epidermis (c.f., Additional file 1: Fig. S6C). As expected, levels of TSLP, an epidermal-generated pro-Th2 cytokine, increased in the skin, but were not detectible in brain (Fig. 5J, K). Finally, circulating IgE levels increased more slowly, becoming significantly elevated only by day 6 (Fig. 3F). Together, these studies illuminate the sequential emergence of cutaneous inflammation, followed by neuroinflammation in VPA-treated mice (Additional file 1: Fig. S8).

### Cutaneous function in ASD humans

The lipid biochemical abnormalities in the brains of VPA-exposed mice mimic well-known abnormalities in the skin of AD patients [78], which in turn provoke abnormalities in permeability barrier function, skin hydration and inflammation [rev. in 40]. Here, we assessed cutaneous function non-invasively in a randomly-selected cohort of young ASD patients (n = 25; mean age = 19) [79], which included patients with and without a prior or concurrent history of AD. Members of the Korean Autism Society gave informed consent to this non-invasive assessment of barrier function, using the same technology as described above for VPA-exposed mice. Consistent with our view that these two disorders are linked, a substantial subset (n = 10) reported a history of prior AD. The ASD patients as a whole appeared to display defects in both permeability barrier function and skin hydration, though the differences did not achieve statistical significance (p = 0.2) (Additional file 1: Fig. S7A, B) [79]. Yet, a substantial subset of these ASD subjects, even without a prior history of skin disease, displayed permeability barrier abnormalities in comparison to age- and gender-matched controls. Thus, many ASD patients with a prior history of AD, and even some without a known history of AD, exhibit abnormalities in cutaneous function. Additional studies would be desirable in human ASD subjects.

## Discussion

Atopic disorders are strongly associated with a subset of ≈ 10% of patients with autism spectrum disorders (ASD) [30, 32, 33], and the severity of the AD phenotype amplifies the prevalence of the AD-ASD association (e.g., [38]). Yet, the mechanistic basis for the link between AD and ASD remains unexplored. Hence, we assessed here the potential basis for the baseline association of AD with ASD in neonatal offspring of pregnant mice, who had been exposed in utero to the anti-seizure medication, valproic acid (VPA) at 12.5 days of fetal age, a standard ASD animal model [6, 56–59]. Pertinently, exposure of pregnant and neonatal humans to potential neurotoxins, like VPA and gabapentin, has been linked to severe hypersensitivity reactions [80–82], and the subsequent development of ASD [6, 83]. The observation of prominent mast cell proliferation and degranulation further supports such an allergic pathogenesis [28]. Although the neuropathology resulting from toxin exposure has been assessed in this mouse model (e.g., [58, 84]), the presence of coexistent cutaneous abnormalities has been missed, likely due to the animals’ furry pélage. Hence, we assessed the impact of VPA in BALB/c mice, where not only brain, but also prominent cutaneous abnormalities were readily apparent at birth. Structural evidence of cytotoxicity in both the brain and the epidermis was further supported by elevated cytokine markers of toxicity and/or chronic inflammation at birth (i.e., TNFα, IL-17A and IFNγ), consistent with a parallel in utero insult to both of these embryologically-linked tissues [41]. Yet, it must be emphasized that studies in mice do not always reflect a similar pathogenic sequence in humans.

Together, these observations suggest that VPA-induced cytotoxicity begins in utero in both fetal epidermis and brain, indicated by elevated cytokine levels of TNFα, IL-17A and IFNγ in both tissues (Fig. 5). Pertinently, elevated Il-17A, TNFα, IL-1α/β have been implicated in the MIA-induced neuroinflammation in ASD [17], and IL-17 helper cells are known to damage cells in the somatosensory cortex [17]. The observation that VPA-exposed, neonatal mice revealed selective cytotoxicity in both the brain and epidermis, with no evidence of injury in other tissues, is consistent with the preferential susceptibility of these two embryologically-linked tissues to a common toxic insult, explaining the ‘baseline’ association of AD with a subset of ASD patients.

The concurrent increase in IFNγ levels not only in the skin, but also in the brain was particularly intriguing, because enhanced cutaneous IFNγ production provokes changes in epidermal ceramide composition that have been proposed to underlie AD [61]. Both the observed decline in bulk sphingolipids, as well as the shift from VLC-FA species towards sphingolipids bearing shorter-chain fatty acids parallels reported IFNγ-induced cutaneous abnormalities, previously proposed to account for the permeability barrier abnormality in AD [85], also accounting for the emergence of ultrastructural features that mimic AD (Additional file 1: Fig. S3). An IFNγ-induced decline in Cer cutaneous production bearing VLC *N*-acyl fatty acids has been further ascribed to cytokine-mediated down-regulation of two fatty acid elongases, ELOVL1 and 4 [75, 85]. Because VPA-exposed brains and skin exhibited a comparable increase in IFNγ levels, this mechanism could explain the concurrent AD-like lipid biochemical alterations in the brains of VPA-exposed animals. Because IFNγ also enhances neurotrophin expression [e.g., nerve growth factor (NGF)], the excessive growth and branching of axons in ASD could also be linked to elevated INFγ levels [86], perhaps compensating for the negative impact of TNFα and IL-17A [87].

A downward shift in FA chain lengths could account, at least in part, for the well-known attenuation of axon myelination in ASD [21]. Shorter-chain FA cannot form the highly-curved structures (Ibid.) that are required to form the membrane bilayers that normally sheath highly-curved axons and dendrites [88–90]. Moreover, dilution of available FA could also be at play—there simply might now be sufficient VLC-FA to coat the vast proliferation in synapse numbers, axon density and axon branching that occurs in ASD.

We next addressed the possibility that the AD phenotype-dependent increase in ASD prevalence could be temporarily linked to the prior emergence of cytokine inflammation in AD, which can appear as early as 1 month of age [76, 77]. Notably, the diagnosis of ASD instead is not firmly established until 18–24 months, though abnormal eye tracking, fMRI findings, excitability, and movement disorders often can be detected much earlier [91]. The initial emergence of very high levels of Th2 cytokines in the skin of VPA-exposed mice, followed only later by the appearance of neuroinflammation, supports a possible skin → brain pathogenic sequence (Fig. 5). Finally, and certainly pertinent to this proposed sequence is our observation that TSLP, the epidermal-derived ‘driver’ of cutaneous Th2 inflammation [76, 77], was readily detected in the skin, but not in the brains of VPA-exposed mice.

If prior changes in skin contribute to downstream neuroinflammation, then cutaneous cytokines must be released into the circulation, followed by their passage across the immature, infantile blood–brain barrier (Fig. 5B). Upon entering the brain, these cutaneous cytokines could then provoke or amplify neuroinflammation [26]. Yet, even 4 days postpartum, the brains of VPA-exposed mice displayed very few inflammatory cells (Additional file 1: Fig. S4), suggesting instead that infiltrating cytokines, particularly the known neurotoxin, IL-17A [27], could directly damage microglia and brain tissues.

## Conclusion

If our proposed AD → ASD sequence holds up to further scrutiny, it is possible that topical formulations, designed to improve barrier function in neonates at risk for AD, could ameliorate or attenuate the downstream concurrent ASD. Pertinently, topical correction of the permeability barrier abnormality alone reduces circulating levels of the three key age-associated cytokines (IL-1β, IL-6, and TNFα) in both aged mice and human skin [54, 55], holding out promise for barrier-corrective therapy in attenuating the subsequent development of ASD. Yet, it also seems reasonable that anti-inflammatory treatments; e.g., with endocannabinoids [56, 58], systemically-administered Mabs, directed against either IL-17A or Th2 cytokines, or bioflavonoids [92–94] could also prevent or ameliorate the downstream development of ASD.

## Supplementary Information

**Additional file 1: Figure S1.** Identification of ASD-like abnormalities in VPA-exposed mice by Morris water maze test: neonatal VPA-exposed mice (7 days of age) were given 4 trials/day to find the target platform, when starting from the four cardinal points around the circumference of the pool. **A** Latency to find the platform in ASD and control mice. **B** Representative searching tracks by swimming mice. **Figure S2.** Demonstration of epidermal permeability barrier abnormality in VPA-exposed mice. Permeability barrier assessment with low molecular weight, water-soluble tracer, lanthanum nitrate (curved arrow depicts outward movement of tracer) leaking into SC interstices, non-curved arrows depict tracer in SC interstices. Mag Bar = 1 µm. **Figure S3.** Basis for impaired cutaneous barrier function in VPA-exposed mice. **A** Normal extracellular bilayers (arrows) in stratum corneum (SC) of Veh-treated mice. **B** Abnormal lamellar bilayer organization (asterisk; open arrows) in VPA-exposed mice. **C** Impaired secretion in VPA-exposed mice, evidenced by a reduction in secreted lamellar body contents at stratum granulosum (SG)-SC interface, as well as entombed (non-secreted) lamellae in SC cytosol (open arrows). **Figure S4.** Representative H&E stained brain sections (400× total magnification) from 1 day (1D) and 4 day (4D) old mice from Veh (**A**, **B**) or VPA (**C**, **D**)—treated female mice. Scattered microglia (white arrows) and neutrophils (yellow arrows) are present within white matter parenchyma. Very few mast cells or other inflammatory cells are observed. **Figure S5.** Sequential changes in additional Th-2 cytokine levels. **A**–**D** Higher levels of TNFα and IL-13 in skin in comparison to brain at birth in VPA-exposed mice. **E**, **F** Comparable levels of IFNγ in skin and brain at birth. **Figure S6. **Immunofluorescence localization of TNFα, IFNγ and Il-13 in skin. **A**–**C** Paraffin-embedded skin sections (5 µm) from 1-day old VPA-and vehicle (Veh)-exposed mice were labelled with rabbit anti-mouse TNFα, IFNγ or IL-13 primary antibodies (vs. no primary antibody), followed by Alexa Fluor 594 (red) conjugated donkey anti-rabbit secondary antibody, and visualized by confocal fluorescence microscopy. DAPI (blue) was used as a nuclear counterstain. Dashed lines indicate dermo-epidermal junction, and solid lines indicate the uppermost layer of the stratum corneum (SC). **D** Quantitation of IFNγ immunostaining in skin of 1-and 4-day-old VPA-vs. vehicle (Veh)-exposed neonates. **Figure S7.** Epidermal functions in ASD subjects: cutaneous barrier function [assessed as transepidermalwater loss (TEWL)] and skin hydration (assessed by corneometry) were measured in 25 ASD volunteers (average age = 19). Though the differences between the three groups as a whole did not reach statistical significance, barrier function and skin hydration declined in a subset of volunteers with ASD, even in absence of concurrent AD or history of prior AD. **Figure S8.** Proposed sequence for barrier-induced provocation of cytokine cascade in AD that could ‘drive’ ASD.

## Data Availability

No datasets were generated or analyzed during the current study.

## Abbreviations

AD: Atopic dermatitis
ASD: Autism spectrum disorder
BBB: Blood–brain barrier
Cer: Ceramide
CNS: Central nervous system
HDAC: Histone deacetylase
IFN: Interferon
IL: Interleukin
LB: Lamellar body
MIA: Maternal immune activation
NGF: Nerve growth factor
SC: Stratum corneum
SG: Stratum granulosum
SD: Standard deviation
SM: Sphingomyelin
TNF: Tumor necrosis factor
TEWL: Transepidermal water loss
TRRAP: Transformation/transcription domain-associated protein
TSLP: Thymic stromal lymphopoietin
VPA: Valproic acid
