# Supplementary material for: Phenotypic overlap between atopic dermatitis and autism
Source: BMC Neurosci. 2021 Jun 22;22:43. doi: 10.1186/s12868-021-00645-0 (PMC8218496; doi:10.1186/s12868-021-00645-0)

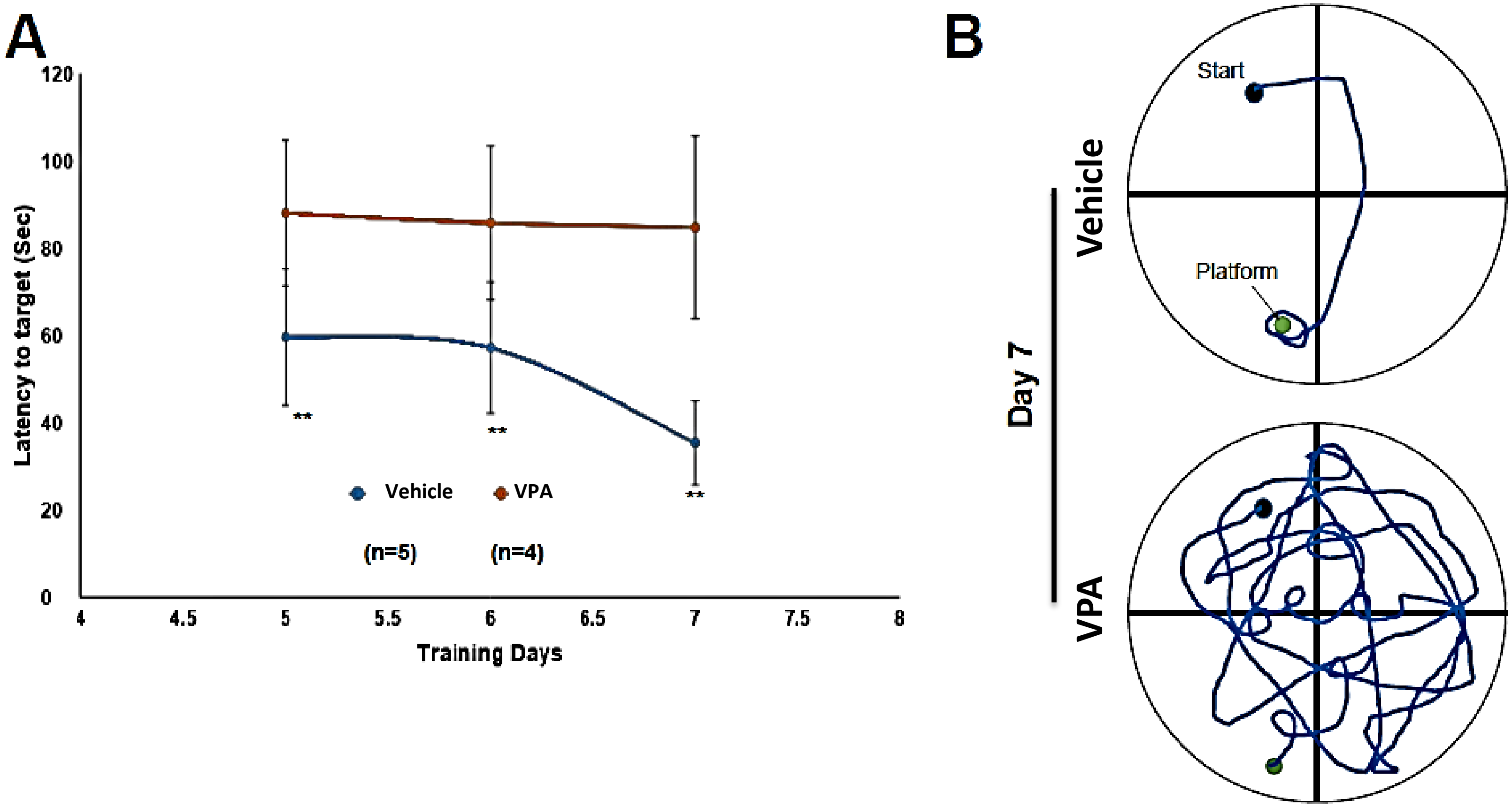

Figure S1: Identification of ASD-Like Abnormalities in VPA-Exposed Mice by Morris Water Maze Test: Neonatal VPA-exposed mice (7 days of age) were given 4 trials/day to find the target platform, when starting from the four cardinal points around the circumference of the pool. **A:** Latency to find the platform in ASD and control mice. **B:** Representative searching tracks by swimming mice.

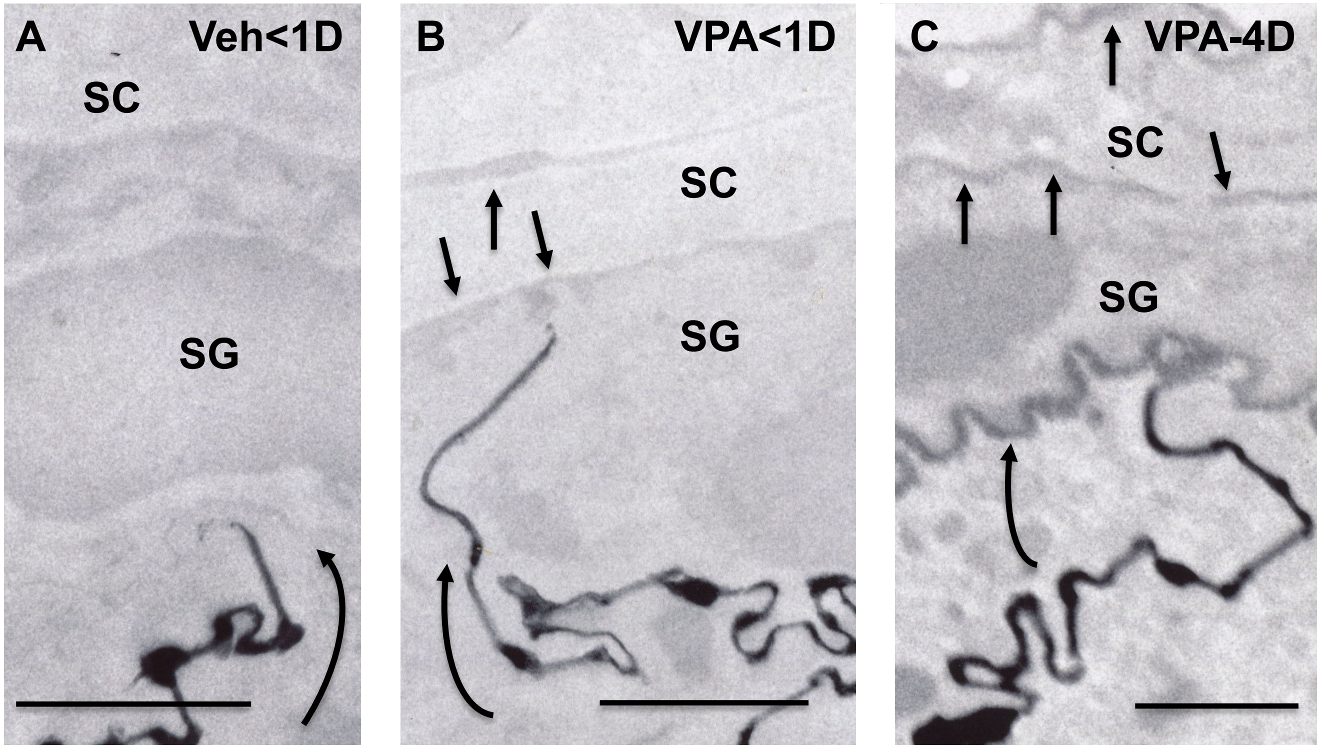

Figure S2: Demonstration of Epidermal Permeability Barrier Abnormality in VPA-Exposed Mice. Permeability barrier assessment with low molecular weight, water-soluble tracer, lanthanum nitrate (curved arrow depicts outward movement of tracer) leaking into SC interstices, non-curved arrows depict tracer in SC interstices. Mag Bar = 1 μm.

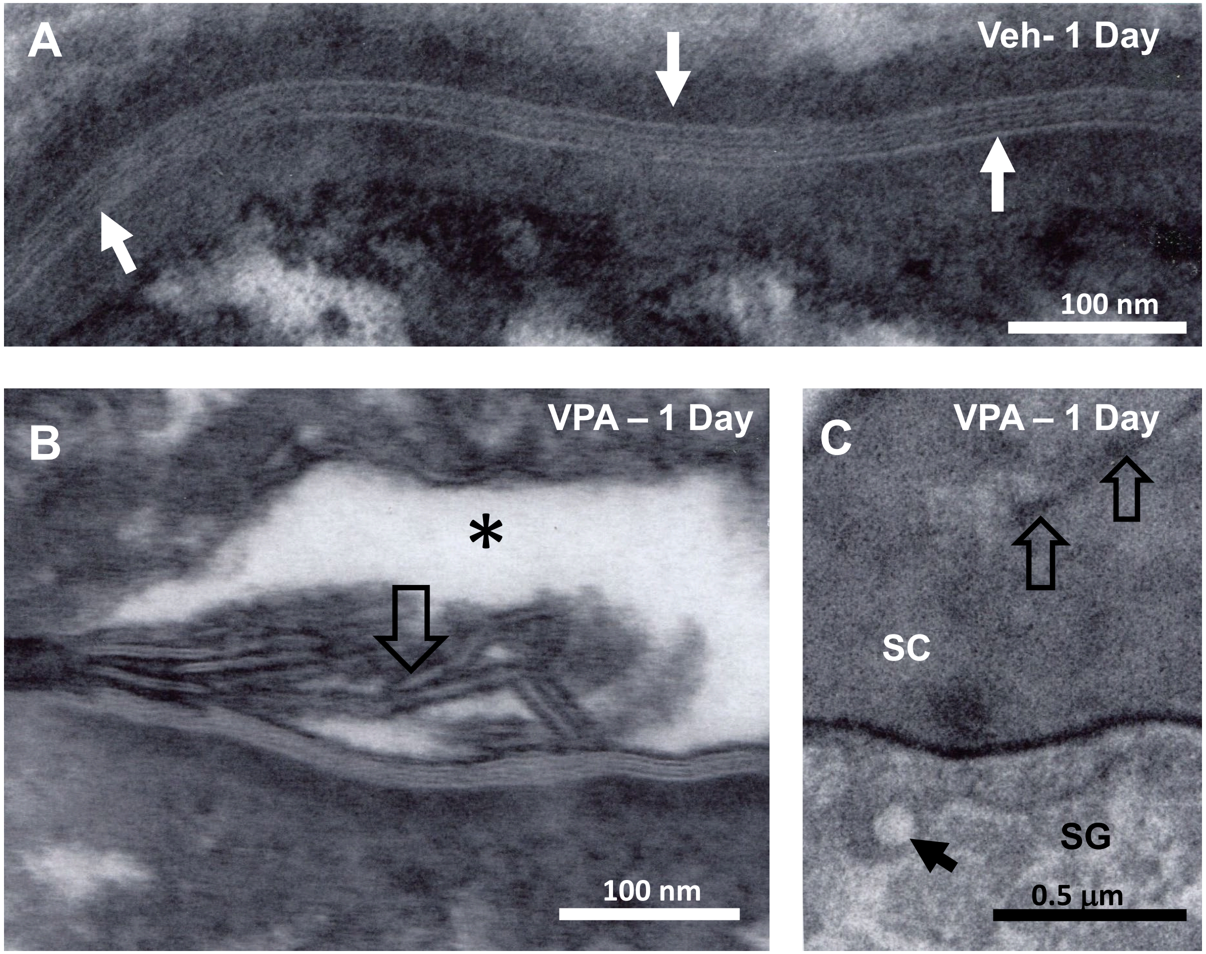

Figure S3: Basis for Impaired Cutaneous Barrier Function in VPA-Exposed Mice. A: Normal extracellular bilayers (arrows) in stratum corneum (SC) of Veh-treated mice. **B:** Abnormal lamellar bilayer organization (asterisk; open arrows) in VPA-exposed mice. **C:** Impaired secretion in VPA-exposed mice, evidenced by a reduction in secreted lamellar body contents at stratum granulosum (SG)-SC interface, as well as entombed (non-secreted) lamellae in SC cytosol (open arrows).

Figure S4: Representative H&E stained brain sections (400X total magnification) from 1 day (1D) and 4 day (4D) old mice from Veh (A&B) or VPA (C&D)-treated female mice. Scattered microglia (white arrows) and neutrophils (yellow arrows) are present within white matter parenchyma. Very few mast cells or other inflammatory cells are observed.

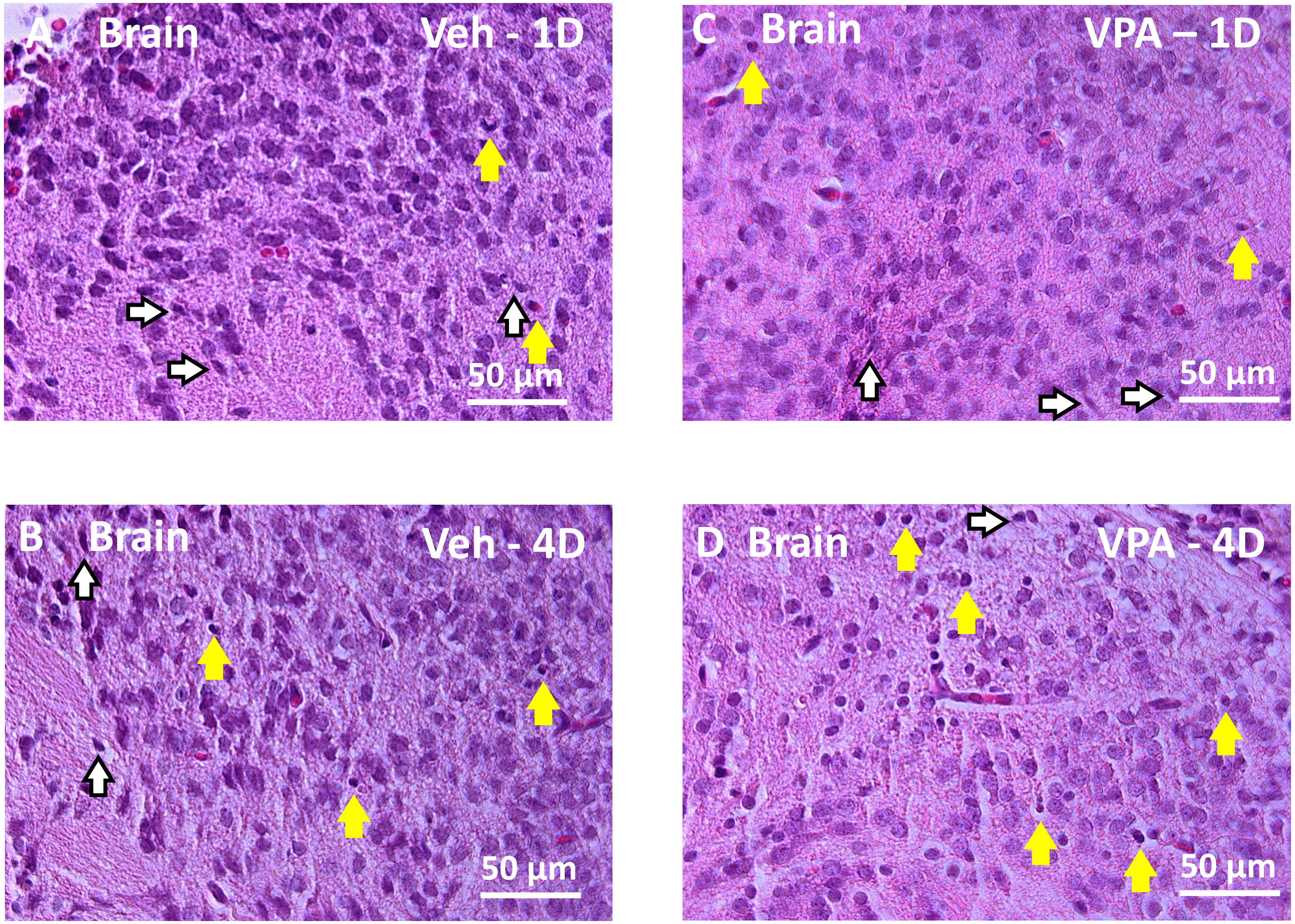

Figure S5: Sequential Changes in Additional Th-2 Cytokine Levels. A-D: Higher levels of TNFα and IL-13 in skin in comparison to brain at birth in VPA-exposed mice. **E+F:** Comparable levels of IFNγ in skin and brain at birth.

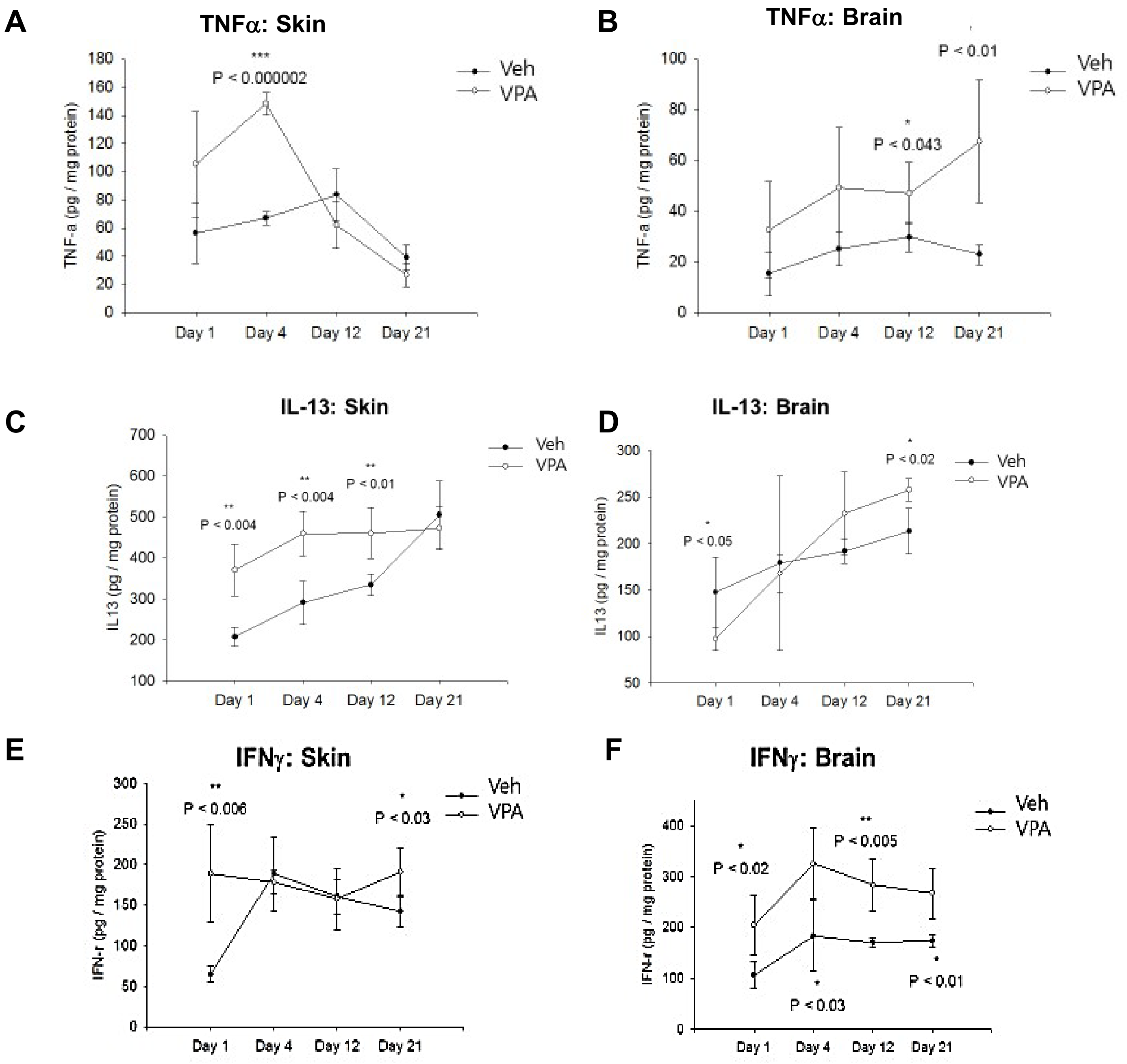

Figure S6: Immunofluorescence Localization of TNFα, IFNγ and Il-13 in Skin. A-C: Paraffin-embedded skin sections (5μm) from 1-day old VPA- and vehicle (Veh)-exposed mice were labelled with rabbit anti-mouse TNFα, IFNγ or IL-13 primary antibodies (vs. no primary antibody), followed by Alexa Fluor 594 (red) conjugated donkey anti-rabbit secondary antibody, and visualized by confocal fluorescence microscopy. DAPI (blue) was used as a nuclear counterstain. Dashed lines indicate dermo-epidermal junction, and solid lines indicate the uppermost layer of the stratum corneum (SC). **D:** Quantitation of IFNγ immunostaining in skin of 1- and 4-day-old VPA- vs. vehicle (Veh)-exposed neonates.

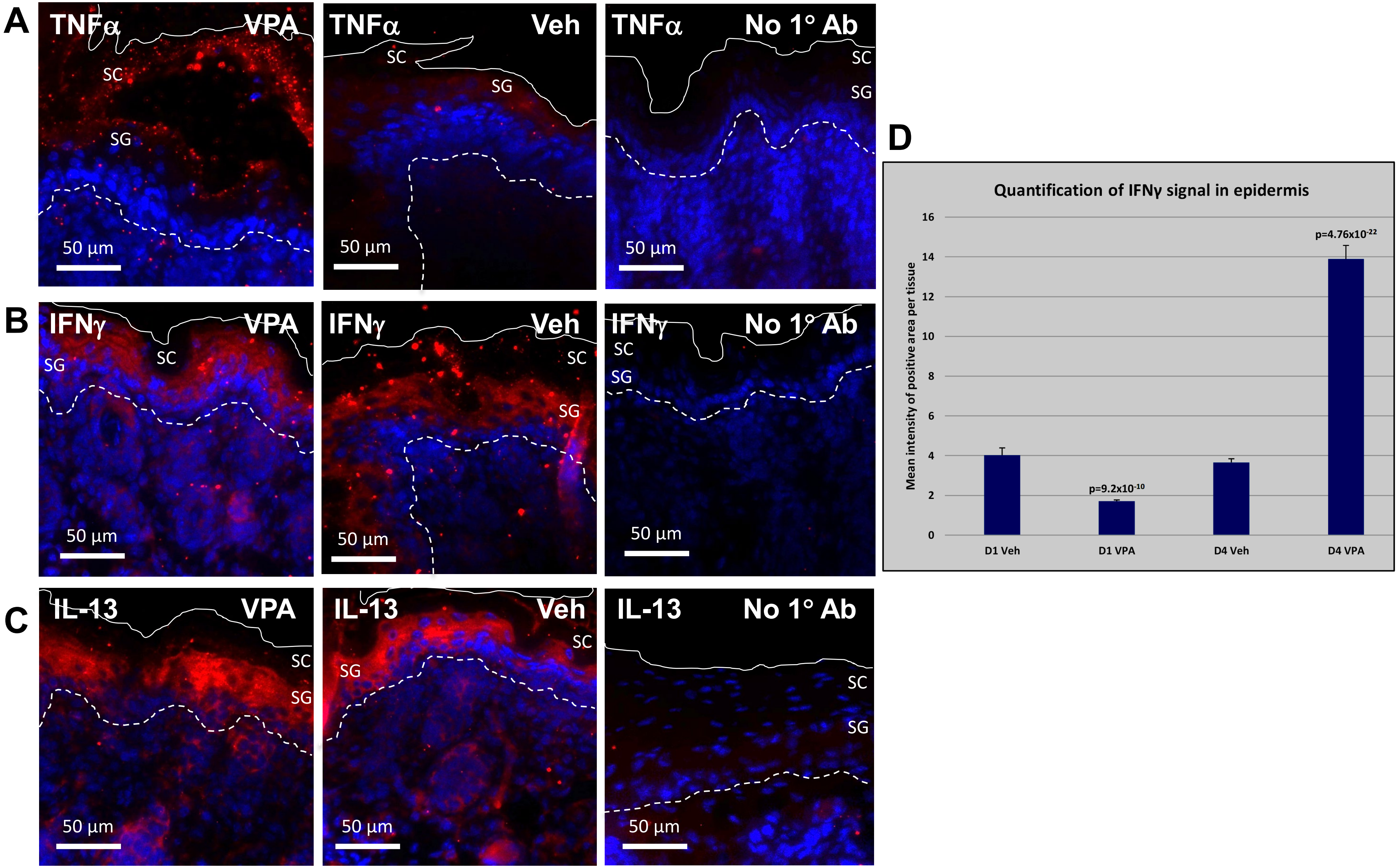

Figure S7: Epidermal Functions in ASD Subjects: Cutaneous barrier function (assessed as transepidermal water loss [TEWL]) and skin hydration (assessed by corneometry) were measured in 25 ASD volunteers (average age = 19). Though the differences between the three groups as a whole did not reach statistical significance, barrier function and skin hydration declined in a subset of volunteers with ASD, even in absence of concurrent AD or history of prior AD.

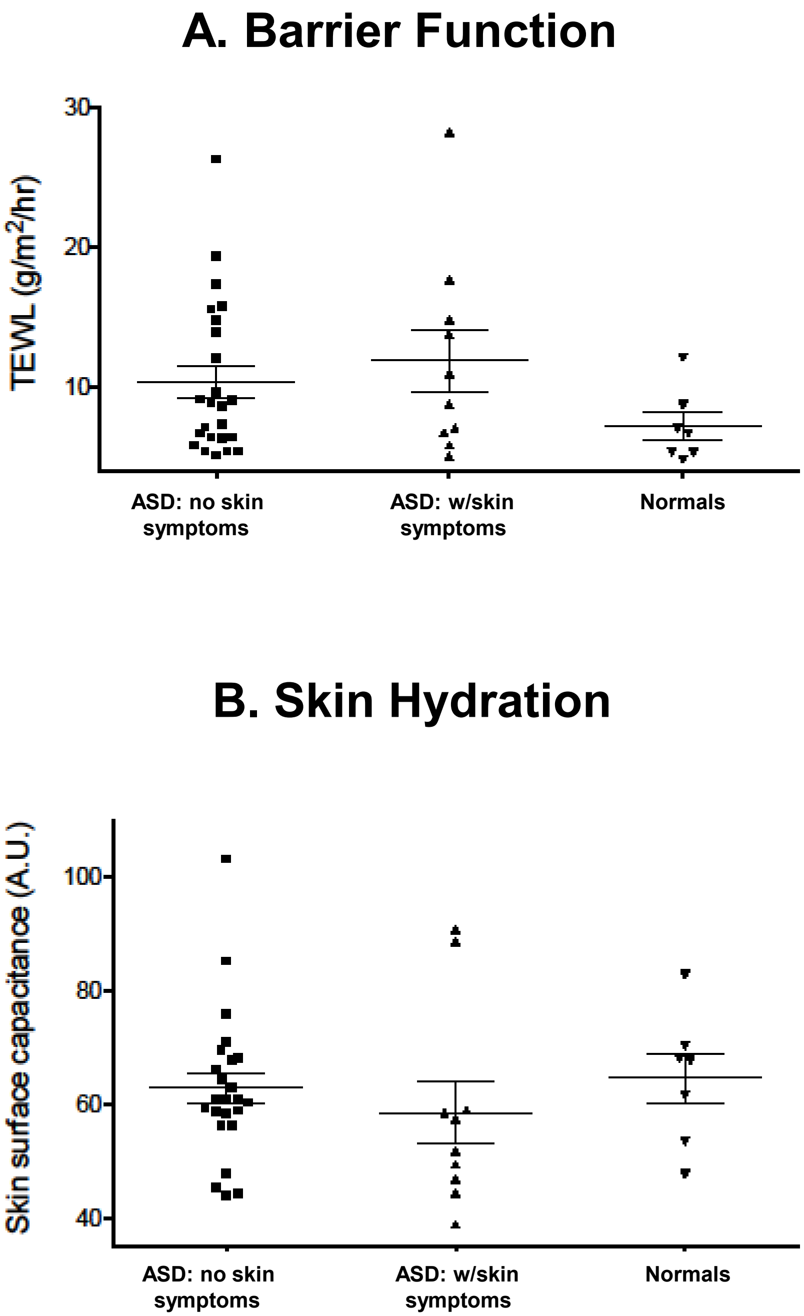

Figure S8: Proposed Sequence for Barrier-Induced Provocation of Cytokine Cascade in AD That Could 'Drive' ASD

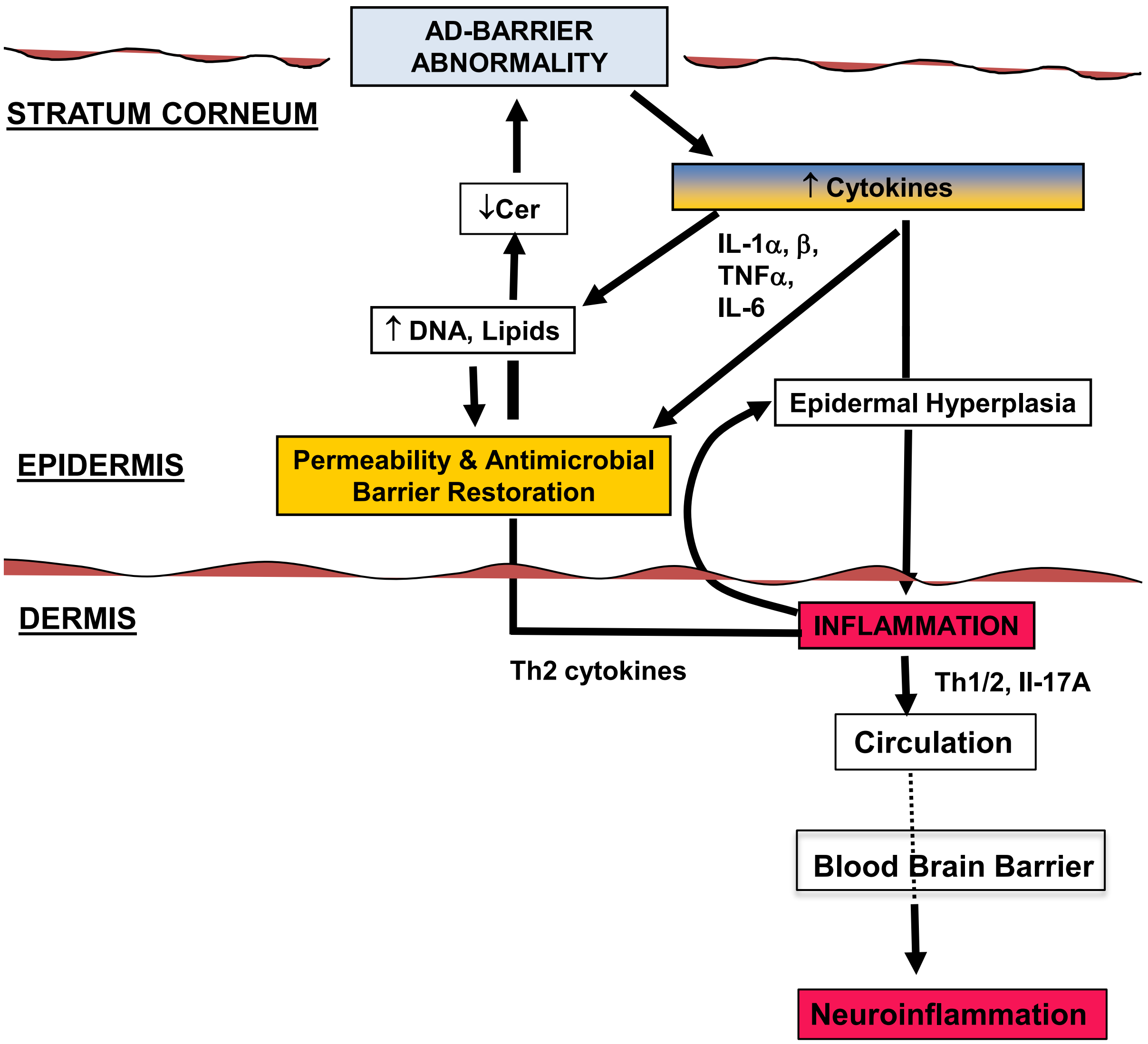

Supplement: Supplementary file 1 — Additional file 1: Figure S1. Identification of ASD-like abnormalities in VPA-exposed mice by Morris water maze test: neonatal VPA-exposed mice (7 days of age) were given 4 trials/day to find the target platform, when starting from the four cardinal points around the circumference of the pool. A Latency to find the platform in ASD and control mice. B Representative searching tracks by swimming mice. Figure S2. Demonstration of epidermal permeability barrier abnormality in VPA-exposed mice. Permeability barrier assessment with low molecular weight, water-soluble tracer, lanthanum nitrate (curved arrow depicts outward movement of tracer) leaking into SC interstices, non-curved arrows depict tracer in SC interstices. Mag Bar = 1 µm. Figure S3. Basis for impaired cutaneous barrier function in VPA-exposed mice. A Normal extracellular bilayers (arrows) in stratum corneum (SC) of Veh-treated mice. B Abnormal lamellar bilayer organization (asterisk; open arrows) in VPA-exposed mice. C Impaired secretion in VPA-exposed mice, evidenced by a reduction in secreted lamellar body contents at stratum granulosum (SG)-SC interface, as well as entombed (non-secreted) lamellae in SC cytosol (open arrows). Figure S4. Representative H&E stained brain sections (400× total magnification) from 1 day (1D) and 4 day (4D) old mice from Veh (A, B) or VPA (C, D)—treated female mice. Scattered microglia (white arrows) and neutrophils (yellow arrows) are present within white matter parenchyma. Very few mast cells or other inflammatory cells are observed. Figure S5. Sequential changes in additional Th-2 cytokine levels. A–D Higher levels of TNFα and IL-13 in skin in comparison to brain at birth in VPA-exposed mice. E, F Comparable levels of IFNγ in skin and brain at birth. Figure S6. Immunofluorescence localization of TNFα, IFNγ and Il-13 in skin. A–C Paraffin-embedded skin sections (5 µm) from 1-day old VPA-and vehicle (Veh)-exposed mice were labelled with rabbit anti-mouse TNFα, IFNγ or IL-13 pr [file 12868_2021_645_MOESM1_ESM.pdf]
